# Carbon Nano-Allotrope/Magnetic Nanoparticle Hybrid Nanomaterials as T2 Contrast Agents for Magnetic Resonance Imaging Applications

**DOI:** 10.3390/jfb9010016

**Published:** 2018-02-06

**Authors:** Yunxiang Gao

**Affiliations:** Department of Chemical and Biomolecular Engineering, Rice University, Houston, TX 77005, USA; yunxiang.gao@rice.edu; Tel: +1-740-591-1917

**Keywords:** MRI, T2-weighted, contrast agent, carbon nanomaterials, carbon nanotubes, graphene, magnetic nanoparticles, drug delivery, theranostic, imaging

## Abstract

Magnetic resonance imaging (MRI) is the most powerful tool for deep penetration and high-quality 3D imaging of tissues with anatomical details. However, the sensitivity of the MRI technique is not as good as that of the radioactive or optical imaging methods. Carbon-based nanomaterials have attracted significant attention in biomaterial research in recent decades due to their unique physical properties, versatile functionalization chemistry, as well as excellent biological compatibility. Researchers have employed various carbon nano-allotropes to develop hybrid MRI contrast agents for improved sensitivity. This review summarizes the new research progresses in carbon-based hybrid MRI contrast agents, especially those reported in the past five years. The review will only focus on T2-weighted MRI agents and will be categorized by the different carbon allotrope types and magnetic components. Considering the strong trend in recent bio-nanotechnology research towards multifunctional diagnosis and therapy, carbon-based MRI contrast agents integrated with other imaging modalities or therapeutic functions are also covered.

## 1. Introduction

^1^H magnetic resonance imaging (MRI), established on measuring the difference of the magnetization properties of hydrogen nuclei, has become one of the most powerful modern imaging tools for non-invasive biomedical diagnostics. In normal states, the hydrogen nuclei of the water or fat molecules in tissue are spinning in random orientations. MRI employs a powerful and uniform external magnetic field to align the randomly-spinning hydrogen nuclei, followed by applying another external radio frequency (RF) to perturb and perpendicularly rotate the spin orientation. By turning off the second RF energy while keeping the first main magnetic field retained, the disturbed spinning hydrogen nuclei return to their resting alignment through various relaxation processes including T1-longitudinal and T2-transverse relaxations, where T1 is the time constant for rotated hydrogen nuclei to re-align with the main external magnetic field, and T2 is the time constant for them to lose the phase coherence caused by the second RF energy. The image contrast in MRI primarily arises from the relaxation time differences of hydrogen nuclei in different environments of the studied tissues. In order to improve the sensitivity and diagnostic confidence, about 30% of the 60 million annually-performed MRI procedures employ chemical contrast agents (CAs) in the investigated area [1]. T1-shortening CAs are mainly based on transition metal ions, such as Gd^3+^ and Mn^2+^, and provide brighter images; whereas T2-shortening CAs usually contain superparamagnetic iron oxide nanoparticles (SPIONs), and produce darker images. The relaxivity parameters r1 and r2 are used to represent the relaxation rate enhancement in unit mM^−1^·s^−1^, which defines the efficacy of the MRI contrast agents.

In recent decades, the blossom of nanotechnology has strongly catalyzed the emergence of innovative functional biomaterials. Quite a few research reviews can be found in the area of biomedical nanomaterial applications, such as nanoparticles (NPs) for Alzheimer’s disease [2], NP contrast agents for molecular imaging [3], magnetic nanomaterials [4], or nanoparticles (MNPs) [5,6] for cancer therapy, as well as quantum dots (QDs) and carbon nanotubes (CNTs) for theranostic applications [7], where ‘theranostic’ describes the medical treatments combining therapy and diagnosis together in one procedure termed as ‘theranosis’ [8]. Specifically, in terms of MRI applications, magnetic nanoparticles need either further functionalization [9] or to be coupled with other nanocarriers [10] for improved biocompatibility, in vivo dispersion, and targeting-delivery capability.

**Figure 1:**
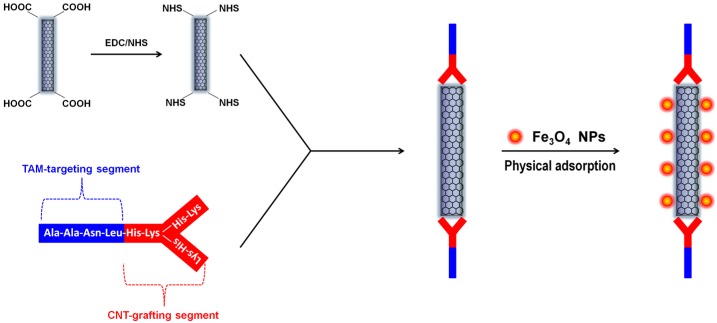
Schematic of a CNT-based T2-weighted MRI contrast agent conjugated with our designed Y-shaped TAM-targeting peptide and loaded with Fe_3_O_4_ nanoparticles via physical adsorption.

Among numerous nano-structured materials, nanoscale carbon allotropes have emerged as highly potential candidates for biomedical applications due to their unique chemical, optical, thermal, optothermal properties, as well as good biocompatibility and low cytotoxicity [11,12,13,14,15]. Hybrid materials containing magnetic components and various carbon nano-allotropes, such as CNTs [16] and nanodiamonds [17] have shown outstanding performances in drug delivery, hyperthermic anti-cancer therapy, and magnetic resonance imaging [18]. For example, we have recently designed and developed a Y-shaped synthetic peptide with the capability of targeting the tumor-associated macrophages (TAMs). The Y-shaped peptide can be either grafted with fluorescein isothiocyanate (FITC) for fluorescence-based bio-imaging or, as shown in Figure 1, grafted onto partially-oxidized carbon nanotubes (OCNTs) via the OCNT’s carboxylic group to form a novel tumor-targeting nanocarrier. The intrinsically-hydrophobic pristine SPIONs can be physically adsorbed onto the unmodified regions of the OCNTs and form SPION-CNT hybrid agents [19]. Intravenous injection of this Y-peptide-*g*-OCNT/Fe_3_O_4_ nano-assembly into 4T1 mammary tumor-bearing mice led to highly-efficient MR imaging of the TAM-infiltrated tumor microenvironment.

**Figure 2:**
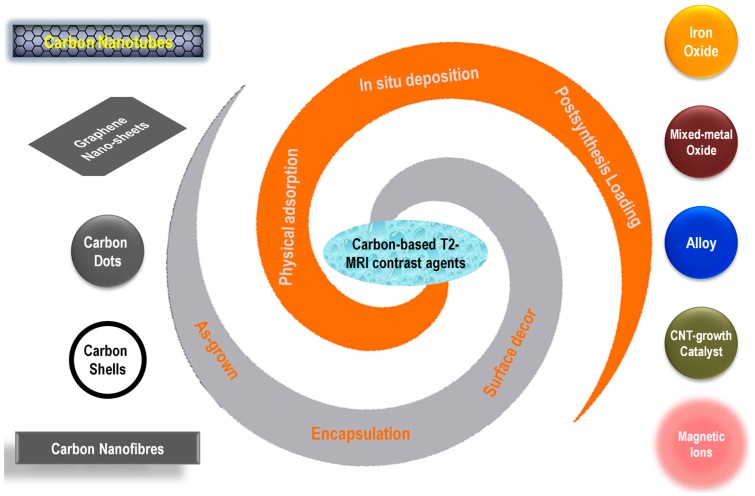
Schematic illustration of various carbon nano-allotropes, magnetic species, and their conjugation strategies used for the preparation of T2-weighted MRI contrast agents.

Recently, new studies on the similar types of carbon-based hybrid MRI CAs are continuously increasing, with the system designs becoming more complex and multifunction-oriented. However, only a limited number of related reviews are available in the literature, either as a brief subsection of a wide topic, or only covering one carbon nanomaterial, such as multi-walled carbon nanotubes [20]. Thus, it is necessary to summarize the recent advances in carbon-based hybrid MRI CAs, especially those published in the past 3–5 years, with a full coverage of various types of carbon nano-allotropes, magnetic components, and the preparation strategies (Figure 2). Since the T1-category positive CAs usually have a better signal-enhancing ability and have been more widely used in clinical diagnosis, and the T2-category negative CAs intrinsically have lower imaging signal intensity, it is a more challenging mission for bio-nanomaterial researchers to explore T2-category CAs, improve their imaging contrast, and expand their multi-functionality. This review will only focus on T2-weighted MRI contrast agents. Other works using various carbon nano-allotropes for diffusion- or T1-weighted MRI applications are representatively selected and listed, but will not be discussed [21,22,23,24,25,26,27,28,29,30,31,32,33,34,35,36,37].

## 2. CNT-MNP Hybrid MRI Contrast Agents

Carbon nanotubes, according to the number of their graphitic layers, can be classified as single-walled carbon nanotubes (SWNTs), double-walled carbon nanotubes (DWNTs), and multi-walled carbon nanotubes (MWNTs), among which SWNTs and MWNTs are the most widely used CNT species due to their relative economic and facile synthesis. CNTs represents one of the primary categories of carbon nano-allotropes that has been widely used for MRI applications.

### 2.1. CNTs with Inherent Magnetic Catalyst Residues

The catalysts used for CNT synthesis are usually magnetic nanoparticles, which after the CNT synthesis, remain physisorbed on the CNTs [38]. It has been demonstrated that the as-grown SWNTs non-covalently functionalized with DNA are potential for MRI CA applications [39]. On the other hand, SWNTs covalently functionalized with carboxylic acid groups (SWNT-COOH) are stable in biological media and cause strong T2 signal decreases in vitro [40]. The r2 relaxivity of SWNT-COOH without any postsynthesis MNP-loadings is as high as 1.54 mM^−1^·s^−1^ at 7 T. Another study also found that, when conjugated with a specific antibody, short SWNTs exhibited very high magnetic resonance r2 relaxivity and could be targeted on breast cancer cells for sensitive MRI detection [41]. 

Similarly, magnetic relaxation behavior was found on catalyst-containing MWNTs. PEGylated MWNTs carrying Fe-based catalyst particles were proved to be efficient T2-weighted MRI contrast agents in vitro [42]. By transplanting MWNT-labeled human islets into the subcutis of rats via a magnetic field and tracking with MRI, Marchetti et al. showed that MWNTs can be an alternative MRI labeling compound to be used with human islets for experimental and transplantation studies [43]. Vittorio et al. found that MWNTs with low metal impurities (2.57% iron from the growth catalyst) can be used as MRI contrast agents for tracking stem cells, which have a significant effect on the T2 transverse relaxation rate of water ^1^H nucleus [44]. Moreover, long Fe nanowires can be formed in the inner cavity of MWNTs under control during the nanotube synthesis process. Through surface functionalization (with folic acids), the MWNTs encapsulating Fe-nanowires can cause a strong decrease in T2 MRI signals by four-fifths, compared to MWNTs without the inner-encapsulated Fe nanowires [45]. Torti et al. explored the dose effect of the magnetic catalyst and found that the in vivo r2 relaxivity of the as-grown MWNTs increased with the mass content of the catalyst within the studied range, which maximized at 2.92% Fe [46]. Another in vitro study considered the effect of Fe dose in a wider range and showed that MWNTs with 10% Fe were less stable, but tended to agglomerate, and the best MRI contrast and biocompatibility was achieved at 2% Fe [47], which is very close to Torti’s results. 

### 2.2. CNTs with Postsynthesis-Loaded Iron Oxide Nanoparticles

As discussed above, the as-grown CNTs usually contain trace amounts (2–3%) of magnetic nanoparticle catalyst, and will not be stable at high MNP contents. On the contrasts, attaching SPIONs on CNTs via postsynthesis methods may allow higher SPION-loading limits. As mentioned previously in the introduction section, simply taking advantage of the physical adsorption, we can load about 20% Fe_3_O_4_ nanoparticles on a TAM-targeting MWNT nanocarrier, which is stable in aqueous solution and gives excellent T2-weighted MRI contrast [19]. Other than physical adsorption, various postsynthesis methods for SPION-loading on CNTs have also been reported. The co-precipitating reaction of ferric and ferrous salts under anaerobic conditions was employed to deposit Fe_3_O_4_ NPs on MWNTs pre-functionalized with poly(diallyldimethylammonium chloride) for liver-targeting MRI applications [48]. Lamanna et al. explored two other approaches for decorating MWNTs with SPIONs: one was based on ligand exchanges between oleic acid-coated SPIONs and oxidized MWNTs, and the other employed a ‘click’ reaction between SPIONs bearing azide groups and functionalized MWNTs bearing alkyne groups. MWNT-SPION hybrid MRI CAs prepared with these chemistries were successfully internalized into tumor cells without showing cytotoxicity, and could be manipulated by a remote magnetic field [49]. Instead of being attached on the outer surface of CNTs, as another choice, SPIONs can be loaded inside CNTs in a postsynthesis manner. Ferrite nanoparticles have been successfully filled in pre-formed MWNTs by introducing an iron stearate precursor into the inner cavity of MWNTs via ultrasonication, followed by a thermo-decomposition [50]. The resulting SPION@MWNT system was proved to be a potential nano-agent for magnetic manipulation of cells, as well as MRI diagnostics. Finally, it is necessary to point out that, for biomaterial researchers working on the development of novel CNT-SPION hybrid MRI contrast agents, the length of CNTs is an important factor to be considered in the material design, because only shortened CNTs can be well-internalized by cells [51,52].

### 2.3. CNTs with Postsynthesis-Loaded Metal Ions, Metal Alloy, and Mixed-Metal Oxide Nanoparticles

Though SPIONs represent a major class of superparamagnetic nanoparticles for T2-weighted MRI CA applications, other types of magnetism sources such as paramagnetic metal ions, alloys, and mixed-metal oxides also received plenty of attention. It was found that even though the as-grown MWNTs-bearing catalyst residues could be used as T2-weighted MRI CAs, further coordinating paramagnetic Fe^3+^ ions on MWNTs pre-grafted with aminophenol ligands would enhance, or even triple, the r2 relativity, making the hybrid agents comparable to the commercial products [53]. Superparamagnetic alloy nanoparticles, such as FePt NPs have excellent stability and magnetic property for T2 negative contrast imaging [54]. Thus, FePt NPs with an average size of 3–4 nm have been deposited on both SWNTs and MWNTs to produce CNT-FePt hybrid CAs for MRI [55]. Magnetic mixed-metal oxide NPs are another class of nanoparticles found application in CNT-based T2-type MRI CAs. MWNT/cobalt ferrite (MWNT-CoFe_2_O_4_) can be prepared via a solvothermal approach, and show an r2 relaxivity of 152.8 mM^−1^·s^−1^ in aqueous solutions at 3.0 T [56]. Co, Ni, Cu, and Zn ferrite oxide (M^II^Fe_2_O_4_) nanoparticles can be deposited on CNTs when the relative precursors are calcinated via a simple microemulsion method [57].

## 3. Graphene-MNP Hybrid MRI Contrast Agents

Graphene, a monolayer 2D material with hexagonally-arranged, tightly-packed, and sp^2^-hybridized carbon atoms in a honeycomb-like lattice, is the basal building block for all graphitic carbon allotropes, including SWNTs and MWNTs. Graphene oxide (GO) nanosheets can be stably dispersed in water or physiological environments. Their excellent biocompatibility and low toxicity make GO a very potential material for biomedical applications. Magnetic reduced-GO (RGO) nanosheets have been successfully synthesized via inverse chemical co-deposition of Fe_3_O_4_ NPs on the reduced GO sheets [58], or the RGO-Fe_3_O_4_ nanocomposites can be prepared via high-temperature decomposition of an iron precursor, iron(III) acetylacetonate (Fe(acac)_3_), in triethylene glycol in the present of RGO nanosheets [59]. Both methods result in efficient T2-type graphene-based MRI CAs. Moreover, Walsh and Hurt et al. showed that graphene oxide can encapsulate Fe_3_O_4_ NPs simultaneously with other nano-probes in the aerosol phase to form nano-cargos for multifunctional biomedical applications, with T2-weighted MRI as one of the integrated functions due to the encapsulated Fe_3_O_4_ NPs [60]. Preparation of the smaller-sized GO-SPIONs, or magnetic graphene quantum dots (MGQDs), can be achieved with a hydrothermal method that simultaneously reduces GO to RGO, deposits Fe_3_O_4_ NPs on the RGO, and shatters the graphene nanosheets to QD size [61]. Liu et al. functionalized RGO-SPION nanocomposites non-covalently with biocompatible polyethylene glycol (PEG) for MRI and multimodal-imaging guided photothermal therapy [62]. Similarly, covalently double-PEGylated RGO-SPIONs show dramatically improved in vivo blood circulation half-life and passive tumor targeting efficacy [63]. 

Except for iron oxides, combining graphene with other magnetic NPs, such as mixed-metal oxides, were also reported. Similar to what has been done on CNT nanocarriers, Wang et al. prepared a graphene oxide/cobalt ferrite (GO-CoFe_2_O_4_) composite through a facile sonochemical method, which showed strong T2-weighted MRI signal enhancement with an r2 relaxivity of 92.71 mM^−1^·s^−1^ at 3.0 T [64].

## 4. MNPs Loaded on Other Carbon Nano-Allotropes

Though graphene and CNTs are the two major carbon allotropes underwent intensive studies for carbon-based MRI CAs, other carbon structures also found their role in this application. Fe-core/C-shell magnetic nanoparticles can be synthesized using a one-step top-down approach, which was conducted between two closely-placed Fe electrodes during the electric-plasma discharge of toluene with the help of an ultrasonic horn [65]. The obtained NPs have improved biocompatibility and stability because of the carbon shell, and can be used as T2-weighted MRI contrast agents with a relaxivity of 70 mM^−1^·s^−1^ in a 7 T MRI scanner. Dai et al. synthesized FeCo-core/single-layer graphitic-carbon shell (FeCo-GC) nanocrystals that were soluble and stable in aqueous solutions. The 7 nm FeCo-GC nanocrystals internalized by mesenchymal stem cells exhibited an r2 relaxivity of 644 mM^−1^·s^−1^, one of the highest values in all reports at 1.5 T, and gave better T2 negative contrasts compared to commercial MRI CAs such as Feridex [66]. Another work reported a complex MNP-core/carbon-shell structure with the magnetic Fe_3_O_4_ nanocrystals clustered in the core and fluorescent carbon dots embedded in the mesoporous carbon shell (Fe_3_O_4_-FCs). The Fe_3_O_4_-FC NPs can be assembled into stable 1D chains in an external magnetic field via the hydrogen bonding effect and π–π stacking between the carbon shells, which show much higher MRI contrast than the individual Fe_3_O_4_-FC NPs [67].

Carbon nanofiber (GNF) is another class of carbon nanostructures seen applications in MRI CAs. Lu et al. have employed graphitized GNFs as nanocarriers for Fe_3_O_4_ NPs. By coating the Fe_3_O_4_-GNF assembly with a SiO_2_ layer, Fe_3_O_4_-GNF@SiO_2_ nanocapsules were prepared for T2-weighted MRI applications [68]. In this work, the hollow GNF was initially filled with iron(III) acetylacetonate, a precursor for the formation of MNPs. Ultra-small Fe_3_O_4_ nanoparticles were then synthesized inside the cavities of GNFs with a high density. The silica coating layer introduces many benefits, including preventing the leakage of the nanoparticles from the GNF hollow cavity, protecting the Fe_3_O_4_ NPs from damage in harsh acidic conditions, and increasing the biocompatibility and stability of the nanocomposites. In another work, Khlobystov et al. performed a detailed study on the controlled preparation of Fe_3_O_4_-GNF nanocomposites [69], in which hollow CNFs acted both as a template and a support for the nucleation and growth of Fe_3_O_4_ NPs. The influence of solvent type and the mass ratio of the magnetite precursor on the structures (size, dispersity, and morphology) and physical properties (magnetization and coercivity) of the Fe_3_O_4_-GNF products were systematically studied. The optimized magnetic hybrid composites were proved to be effective T2 negative MRI contrast agents with an excellent transverse relaxivity of 268 ± 13 mM^−1^·s^−1^ at 9.4 T, which surpasses most commercial MRI materials or state-of-the-art nanoscale CAs performing under such a high magnetic field.

## 5. Carbon-MNP Hybrid MRI Contrast Agents Integrated with Multiple Functions

Although MRI is the most powerful tool for deep penetration and high-quality 3D imaging of tissues with anatomical details, its sensitivity is not as good as that of the radioactive or optical methods. The application of multi-modality imaging probes could provide more accurate information in clinical settings. For example, fluorescence imaging is highly sensitive and has potential for real-time imaging, but it has limited depth perception; computed tomography (CT) imaging offers accurate and bright images; positron emission tomography (PET) uses signals emitted by compounds labeled with positron-emitting radioisotopes for bio-imaging. Combining the deep perception of MRI with other, more sensitive, imaging modals, the carbon-based T2 MRI contrast agents can provide highly accurate and detailed information associated with diseases. Indeed, plenty of efforts have been made to integrate multimodal bio-imaging techniques, such as fluorescence imaging, Raman imaging, microwave-induced thermoacoustic imaging (TAI), CT, PET, and single-photon emission computed tomography (SPECT) on the carbon-based T2 MRA CAs, especially in the past 3–5 years.

Moreover, advanced clinical technologies are attempting to combine therapy and diagnosis functions together in one treatment for so-called “theranosis” applications. The therapy methods to be integrated include controlled drug delivery, targeted drug delivery, and hyperthermia (or thermotherapy). Hyperthermia exposes tumor tissues to high temperatures and kills cancer cells with minimal injury to the normal tissues. 

**Figure 3:**
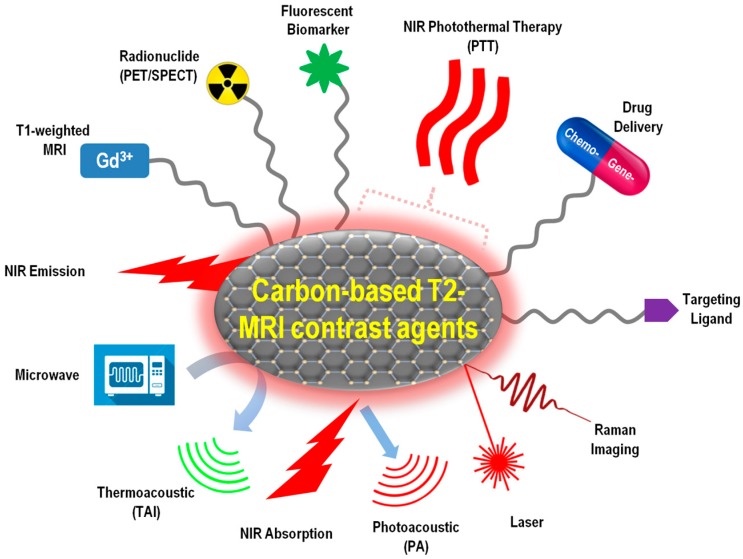
Schematic illustration of T2-type MRI contrast agents integrated with multiple theranostic functions.

In this section, representative new progress in carbon-MNP hybrid T2-type MRI CAs with integrated multimodal imaging capability, drug delivery, and hyperthermia functions will be summarized (Figure 3). All the carbon-based nanocomposites discussed in this section have MRI as a default function, and possess at least one more other function for either diagnosis or therapy purposes.

### 5.1. Carbon-MNP Hybrid MRI Agents with both T1 and T2 Contrast

Although most gadolinium-based CAs are for T1-weighted MR imaging, recent studies have shown that gadolinium-loaded ultra-short SWNTs (GNTs) can be used as high-performance MRI CAs with both excellent T1 and T2 contrasts [70]. GNTs were prepared by encapsulating Gd^3+^ ion clusters within the hollow cavity of SWNTs. The prepared GNTs not only outperform the traditional Gd^3+^-chelate MRI CAs in r1 relaxivity by about 40 times at clinical field strengths, but also show strong T2-weighted MRI contrast. Other works also reported similar findings that Gd-SWNT nano-agents in solution have both high r1 and r2 relaxivity at either very low (0.01 MHz) or clinically-relevant (61 MHz) magnetic fields [71]. GNTs suspended in 1% Pluronic F127 solution gave a high r2 value of 578 mM^−1^∙s^−1^ at 9.4 T with excellent T2 negative contrast in vivo [72]. For Gd^3+^-functionalized nanodiamond, Meade et al. found that the dose of the loaded Gd^3+^ had significant influences on T2-weighted MRI signals. When the cellular concentration of Gd^3+^ exceeds a certain threshold, positive contrast diminishes to yield negative contrast, and T2 negative contrast becomes a predominant effect over T1 at the highest Gd^3+^ dose [73]. However, there are opposite cases in which Gd^3+^-functionalized MWNT nano-agents were studied, and it was found that the T2 effect was independent of Gd^3+^, but exclusively related to the presence of MWNTs [74]. 

Other than Gd^3+^-functionalization, Mn^2+^-CNTs were also prepared and shown both T1 and T2 effects. SWNTs functionalized with polydopamine, and further modified by PEG chelate Mn^2+^ ions to offer both T1- and T2-weighted contrasts for MR imaging [75].

As for non-ionic magnetism sources, LaF_3_:Eu:Gd nanocrystals have been coated on MWNTs to simultaneously provide both powerful T1- and T2-weighted signals for MR imaging [76]. The FeCo-GC hybrid nanocrystals reported by Dai et al. and reviewed in Section 4 of this review as a T2-type contrast agent show very high r1 relaxivity, as well, which enables both T1 positive-contrast and T2 negative-contrast [66].

### 5.2. Carbon-MNP Hybrid MRI Contrast Agents with Multimodal Imaging Capability

Multi-modal imaging provides multi-aspect, more accurate and complete information for biomedical diagnosis and therapy. Strano et al. demonstrated that the as-grown SWNTs containing residue-amounts of magnetic catalysts could be utilized as a multimodal bio-imaging probe due to their intrinsic near-infrared fluorescence and Raman scattering, in addition to the pre-existing MRI function established on their superparamagnetic catalysts [39]. Unlike SWNTs, MWNTs intrinsically do not emit fluorescence. However, PEGylated MWNT can be stained with external fluorescent organic dyes such as monodansylcadaverine (MDC), diamidino-2-phenylindole (DAPI), or fluorescein isothiocyanate (FITC) to achieve MRI-fluorescence bimodal cellular imaging [42,55,77]. On the other hand, carbon QDs emitting violet fluorescence at 320 nm excitation can be used as an intrinsic source of photoluminescence. These QDs have been embedded in GO-Fe_3_O_4_ probes for MRI-fluorescence dual-modality imaging [61]. Similarly, another work conjugated carboxyl-terminated graphene quantum dots (GQD) on the outer surface of a Fe_3_O_4_-core/SiO_2_-shell hybrid system. The Fe_3_O_4_@SiO_2_@GQD hybrid nanoparticles functionalized with cancer-targeting molecule folic acid can be used for MRI-fluorescence duo-imaging of living Hela cells in vitro [78]. 

Liu et al. conjugated protamine with PEG-functionalized SWNTs and prepared an MRI CA exhibiting extremely efficient cell-entry into human mesenchymal stem cells without affecting their proliferation and differentiation. In addition to their inherent MRI capability, the strong Raman scattering from SWNTs enables ultrasensitive Raman imaging of as few as 500 stem cells administrated into mice [79]. The same research group later developed a multifunctional nano-platform based on PEGylated, mesoporous, silica-coated SWNTs, which could serve as a multimodal imaging probes simultaneously enabling MR, photoacoustic, Raman, and NIR imaging [80]. Microwave-induced thermoacoustic imaging (TAI) maps the microwave absorption distribution of the studied targets. Since MWNTs are outstanding electric absorption materials, while their magnetic absorption is negligible, they have been used to enhance the TAI signal of the tissue as a contrast to de-ionized water and blood [45]. Moreover, incorporating radioactive elements is an efficient way to introduce PET imaging capability. Double-PEGylated RGO-SPION nanoparticles labeled with ^64^Cu show multimodality in MR, PET, and photoacoustic imaging due to the strong superparamagnetism from SPIONs, radioactivity from ^64^Cu, and NIR absorbance from RGO [63]. Al-Jamal et al. designed another radio-labeled probe based on MWNT-SPION hybrid systems, where γ-Fe_2_O_3_ NPs were deposited in situ on the oxidized sites of MWNTs, and the radiolabel ^99m^Tc were introduced via a functionalized bisphosphonate to enable 3D whole body SPECT/CT imaging and quantitative organ bio-distribution profiling in mice [81].

### 5.3. Carbon-MNP Hybrid MRI Contrast Agents with Drug Delivery Capability

Carbon-based MRI CAs integrated with drug delivery capability represents a new promising trend in nanomedicine research. A popular anti-cancer drug, doxorubicin (DOX), has been loaded onto an MWNT-CoFe_2_O_4_ hybrid system and achieved sustained and pH-responsive DOX release [56]. Similarly, the GO-CoFe_2_O_4_ composite prepared by a sonochemical method showed a high DOX loading capacity of 1.08 mg/mg and a sustained, PH-responsive drug releasing behavior [64]. More carbon-based MRI CAs with controlled DOX delivery function are reported on antibody-conjugated magnetic SWNTs [82,83] and GO-SPION systems [84]. Another anti-tumor model drug, 5-fluorouracil, was loaded onto the surface of RGO-Fe_3_O_4_ nanocomposites with a high loading capacity and showed pH-dependent release [58]. The colorectal cancer treatment drug, oxaliplatin, was encapsulated into the CNT cavity of a PEGylated MWNT-SPION MRI probe, which allowed sustained oxaliplatin release up to 96 hours with the MRI monitoring option [85]. Anesthetic lidocaine hydrochloride (LH) was loaded on RGO-SPION quantum dots through π–π stacking between the aromatic rings of LH and the RGO surfaces, and a steady, full drug release from the QDs was observed over 8 h [61]. 

In addition to delivering traditional chemo-drugs, small interfering RNA (siRNA)-based gene delivery has become a new trend for cancer therapy in recent years [86]. Zhang et al. modified MWNT-Fe_3_O_4_ CAs with polyethyleneimine (PEI) and polyethylene glycol (PEG) to improve their solubility and biocompatibility. Then, human telomerase reverse transcriptase (hTERT) siRNA was adsorbed on the nanocarrier surface via electrostatic interaction. The obtained hybrid system MWNT-Fe_3_O_4_-PEI-PEG/siRNA efficiently delivered siRNAs into MCF-7 human breast cancer cells and significantly inhibited tumor cell growth via gene silencing, which can be monitored via the MRI function from the same agent [87].

### 5.4. Carbon-MNP Hybrid MRI Contrast Agents with Hypothermia Capability

In complement to chemotherapy and gene therapy, photothermal therapy (PTT) is another powerful technique that can be integrated on various carbon-based MRI CAs. Taking advantage of the broad NIR absorption of graphene, photosensitizer of any wavelength within 600–1200 nm will facilitate the light-induced phototherapy. Lin et al. adopted a simple non-covalent approach to immobilize a hydrophobic photosensitizer, silicon napthalocyanine bis (trihexylsilyloxide) (SiNc_4_), onto water-dispersible SPION-and-fluorescein-decorated graphene nanosheets via π–π stacking to yield an MRI-fluorescent dual-imaging probe with PTT capability [88]. The FeCo-GC hybrid nanocrystals reported by Dai et al. showed NIR absorbance at 808 nm and, thus, afforded the conversion of NIR photon energy into thermal energy for PTT applications [66]. Liu et al. utilized both the intrinsic high NIR optical absorbance of RGO and the strong magnetic property of SPIONs, and designed an efficient theranostic agent for in vivo MRI-guided PTT treatment. This RGO-based MRI contrast agent allows ultra-efficient tumor ablation using a laser power density as low as 0.5 W·cm^−2^ [62]. On a similar system, the same research group also loaded Au nanoparticles to further enhance the thermo-ablation capabilities of the nanocomposites [89]. Many other MRI CA systems based on RGO-SPIONs, CNT-SPIONs, and CNT-FePt have also been studied for simultaneously diagnosing and photothermal cancer treatments [50,55,90,91]. RGO-SPION quantum dots [61] and the fluorescent carbon dots embedded in the mesoporous carbon shell of the Fe_3_O_4_-FC MRI CAs [67] also efficiently convert NIR light to heat, and can be used for photothermal ablation of tumor cells under near-infrared irradiation.

## 6. Conclusions and Future Perspectives

To summarize, carbon nano-allotropes are highly promising nano-carriers for the next-generation of MRI contrast agents. The main advantage lies in their relative low bio-toxicity, versatile surface modification strategies, unique structural cavities, and intrinsic NIR absorption/emission. This review covers the most recent research progresses in carbon-based T2-shortening MRI contrast agents, especially those published in the past 3–5 years. In terms of the allotrope species, a wide variety of novelly-structured carbon nanomaterials including single-walled/multi-walled carbon nanotubes, graphene and reduced graphene oxide nano-sheets, graphene quantum dots, carbon dots, carbon nanofibers, single-layer graphitic carbon nano-shells, and mesoporous carbon shells have all found their unique roles as efficient nano-carriers for MRI contrast agents. In terms of the magnetism origins, various magnetic species, such as iron oxide nanoparticles, mixed-metal oxide nanoparticles, metal alloys, and magnetic ions, have been successfully coupled with carbon nanomaterials for enhanced MR imaging. The coupling methods have versatile options from inner encapsulation to surface decoration, from in situ deposition to physical adsorption, and from real-time generation during the formation of the carbon structures to postsynthesis modification. Such rich possibilities in choosing the hybrid components and their combinations, as well as the versatile preparation strategies, endow the previous single-functional T2-shortening MRI contrast agents multiple functions. Due to the intrinsic near-infrared absorption and fluorescence of many carbon nanomaterials, NIR imaging and NIR-triggered photothermal therapy are the most commonly seen add-in functions to the carbon-based T2-type MRI contrast agents. Other imaging modals that have been successfully integrated include T1-weighted MR, bio-marker fluorescence, Raman, CT, PET, SPECT, thermoacoustic, and photoacoustic imaging. As for therapeutic functions, NIR-triggered hypothermia, drug-delivery-based chemotherapy, and siRNA-delivery-based gene therapy have also been successfully integrated, turning the carbon-based MRI contrast agents into more powerful all-in-one theranostic tools.

Although the carbon nano-allotrope/magnetic nanoparticle hybrid nanomaterials have been extensively studied for MRI applications, many more future efforts are still expected, especially in achieving better-defined and well-controlled parameters, such as agent size for real clinical setting applications. Moreover, humans’ ever-growing ambition in theranostic techniques requires more complex all-in-one nano-agents for challenging medical tasks. Developing novel carbon-based MRI agents with the capability of dynamically tracking pathological changes, or stimuli-responsively applying medical treatments, can potentially be significant for future studies.

## Abbreviation

Abbreviation	Label
CA	Contrast agent
CNF	Carbon nanofibre
CNT	Carbon nanotube
CT	Computed tomography
DAPI	Diamidino-2-phenylindole
DOX	Doxorubicin
DWNT	Double-walled carbon nanotube
FC	Fluorescent carbon
FITC	Fluorescein isothiocyanate
GC	Graphitic carbon
GNT	Gadolinium-loaded CNT
GO	Graphene oxide
GQD	Graphene quantum dot
hTERT	Human telomerase reverse transcriptase
LH	Lidocaine hydrochloride
MCF-7	Michigan Cancer Foundation-7
MDC	Monodansylcadaverine
MGQD	Magnetic graphene quantum dot
MNP	Magnetic nanoparticle
MR	Magnetic resonance
MRI	Magnetic resonance imaging
MWNT	Multi-walled carbon nanotube
NIR	Near-infrared
NP	Nanoparticle
OCNT	Oxidized carbon nanotube
PEG	Polyethylene glycol
PEI	Polyethyleneimine
PET	Positron emission tomography
PTT	Photothermal therapy
QD	Quantum dot
RF	Radio frequency
RGO	Reduced graphene oxide
RNA	Ribonucleic acid
si-RNA	Small interfering RNA
SPECT	Single-photon emission computed tomography
SPION	Superparamagnetic iron oxide nanoparticle
SWNT	Single-walled carbon nanotube
SWNT-COOH	SWNTs grafted with carboxylic acid groups
TAI	Thermoacoustic imaging
TAM	Tumor-associated macrophage
